# Controversies in the management of HIV-positive adults and review of the literature - a case report

**DOI:** 10.1186/1471-2334-14-S4-P47

**Published:** 2014-05-29

**Authors:** Manuela Arbune, Anca Arbune

**Affiliations:** 1 “Dunărea de Jos” University, Galați, Romania

## 

Major HIV/AIDS organizations provide resistance testing guidelines, but the recommendation in chronically infected and treatment-naïve patients is controversial. We present a case including HIV resistance reports and review of the literature.

A 27 year old man was diagnosed with HIV 8 years ago, when nadir CD4 was 5/cmm. He experienced 3 antiretroviral combinations with reverse-transcriptase nucleosides inhibitors and protease inhibitors. Although he is adherent and his immunity was improved, undetectable HIV-RNA was never achieved. In January 2013 he presented with increasing viral load to 14,228 copies/mL and decreasing immunity more than 10%. The genotyping resistance testing assay was performed on HIV-RNA. A large number of genotyping mutations were found, significant for resistance to most nucleoside reverse transcriptase inhibitors and protease inhibitors. Although the patient has never been exposed to non-nucleoside reverse transcriptase inhibitors, the mutation K103 was also present. The review of patient's history revealed HIV positive ex-wife who died in 2008. She had received treatment with nevirapine as part of one from seven antiretroviral experienced combinations. An identical pattern of genotyping resistance with the isolate of our patient, including the mutation K103, was revealed. His present wife is HIV negative and she is planning to become pregnant, but recommendation of pre-exposure prophylaxis is controversial.

Large use of non-nucleoside inhibitors in Romania justify routine resistance testing of new HIV infected patients, especially if they are partners of antiretroviral experienced patients.

